# BGP-15 Inhibits Hyperglycemia-Aggravated VSMC Calcification Induced by High Phosphate

**DOI:** 10.3390/ijms22179263

**Published:** 2021-08-26

**Authors:** Annamária Nagy, Dávid Pethő, Rudolf Gesztelyi, Béla Juhász, György Balla, Zoltán Szilvássy, József Balla, Tamás Gáll

**Affiliations:** 1 Division of Nephrology, Department of Medicine, Faculty of Medicine, University of Debrecen, 4032 Debrecen, Hungary; nagy.annamari90@gmail.com (A.N.); petho.david@med.unideb.hu (D.P.); gall.tamas@med.unideb.hu (T.G.); 2 Kálmán Laki Doctoral School, Faculty of Medicine, University of Debrecen, 4032 Debrecen, Hungary; 3 Department of Pharmacology and Pharmacotherapy, Faculty of Medicine, University of Debrecen, Nagyerdei krt 98, 4032 Debrecen, Hungary; gesztelyi.rudolf@pharm.unideb.hu (R.G.); juhasz.bela@pharm.unideb.hu (B.J.); szilvassy.zoltan@med.unideb.hu (Z.S.); 4 ELKH-UD Vascular Biology and Myocardial Pathophysiology Research Group, Hungarian Academy of Sciences, University of Debrecen, 4032 Debrecen, Hungary; balla@med.unideb.hu; 5 Department of Pediatrics, Faculty of Medicine, University of Debrecen, 4032 Debrecen, Hungary

**Keywords:** BGP-15, vascular smooth muscle cell, vascular calcification, high glucose, diabetes mellitus

## Abstract

Vascular calcification associated with high plasma phosphate (Pi) level is a frequent complication of hyperglycemia, diabetes mellitus, and chronic kidney disease. BGP-15 is an emerging anti-diabetic drug candidate. This study was aimed to explore whether BGP-15 inhibits high Pi-induced calcification of human vascular smooth muscle cells (VSMCs) under normal glucose (NG) and high glucose (HG) conditions. Exposure of VSMCs to Pi resulted in accumulation of extracellular calcium, elevated cellular Pi uptake and intracellular pyruvate dehydrogenase kinase-4 (PDK-4) level, loss of smooth muscle cell markers (ACTA, TAGLN), and enhanced osteochondrogenic gene expression (KLF-5, Msx-2, Sp7, BMP-2). Increased Annexin A2 and decreased matrix Gla protein (MGP) content were found in extracellular vesicles (EVs). The HG condition markedly aggravated Pi-induced VSMC calcification. BGP-15 inhibited Pi uptake and PDK-4 expression that was accompanied by the decreased nuclear translocation of KLF-5, Msx-2, Sp7, retained VSMC markers (ACTA, TAGLN), and decreased BMP-2 in both NG and HG conditions. EVs exhibited increased MGP content and decreased Annexin A2. Importantly, BGP-15 prevented the deposition of calcium in the extracellular matrix. In conclusion, BGP-15 inhibits Pi-induced osteochondrogenic phenotypic switch and mineralization of VSMCs in vitro that make BGP-15 an ideal candidate to attenuate both diabetic and non-diabetic vascular calcification.

## 1. Introduction

Diabetes mellitus (DM) is one of the most prevalent chronic diseases with significant morbidity and mortality. Having stated by the Global Burden of Disease (GBD) report for 2015, the prevalence of DM increased from 330 million persons in 2005 to 435 million in 2015 [1] with a significant rise of annual deaths from DM [2]. Long-term complications of DM include diabetic retinopathy, neuropathy, nephropathy, and cardiovascular diseases.

Vascular calcification (VC) is a frequent complication of DM [3,4]. Intimal calcification is commonly associated with atherosclerosis, while medial calcification mainly develops in patients with DM [5] and chronic kidney disease [6]. Medial calcification is a significant independent predictor of cardiovascular mortality in diabetic patients [7].

Pathophysiologically, VC is an active, finely tuned process that resembles bone formation in many aspects [8]. Calcifying stimuli, among them high phosphate (Pi) [9], hypercholesterinemia, and elevated Lipoprotein(a) blood level [10] induce a phenotypic trans-differentiation of vascular smooth muscle cells (VSMCs) into osteoblast-like cells, facilitate the deposition of calcium phosphate crystals in the extracellular matrix (ECM) and the release of calcifying vesicles [11], representing an imbalance between calcification inducers and inhibitors [12]. In addition, extracellular vesicles (EVs) are important contributors to the development of several diseses such as cardiovascular disease [13,14], diabetes [15], as well as cancer [16]. High serum Pi levels are associated with coronary artery calcification not only in chronic kidney disease [17] but also even in patients with normal renal function [18] underlining the key role of Pi in VC. Dietary Pi restriction markedly reduces mortality in uremic rats with VC [19], while high Pi diet induces vascular mineralization in nephrectomy rat model [20]. Owing to the growing amounts of Pi additives in the food, there may be a link between dietary Pi and VC. However, the direct connection between the high Pi intake and adverse clinical outcomes needs to be further supported in future human studies.

Over the past years, hard evidence shows that hyperglycemia and pathologic glucose homeostasis dramatically accelerate VC [21,22]. Hyperglycemia and diabetes mellitus aggravate vascular calcification and osteochondrogenic transformation of VSMCs [3]. Diabetic kidney disease develops in approximately 30% of people with type 1 diabetes and 50% of people with type 2 diabetes [23], when it progresses to end-stage kidney failure, high plasma concentration of plasma Pi joins to the pathologic glucose hemostasis as an additive risk factor for vasculopathy [24]. In addition, oxidative stress and low-grade chronic inflammation are also contributors to vascular calcifications in diabetes, both of which are enhanced by a state of dysglycemia [25,26].

Krüppel-like factor 5 (KLF-5) plays a vital role in VSMCs’ osteoblastic conversion. In response to high Pi, KLF-5 expression increases followed by the loss of SMC-specific markers and the parallel induction of osteochondrogenic genes [27]. Downregulation of smooth muscle-specific markers such as smooth muscle α-actin (ACTA) is a common feature of VSMC calcification [28]. Upregulation of genes involved in osteoblast differentiation and bone formation such as Transcription factor Sp7 (Sp7; also known as Osterix) [29], Homeobox protein MSX-2 (Msx-2) [30], and Bone morphogenetic protein-2 (BMP-2) [31] have key roles in VSMCs calcification. In addition, recent evidence has shown that pyruvate dehydrogenase kinase-4 (PDK-4), a key enzyme for the regulation of glucose oxidation via inhibition of the pyruvate dehydrogenase complex, has a strong pro-calcifying role in vascular calcification [32,33].

BGP-15 (C14H22N4O2·2HCl) is a nicotinic amidoxime derivate, (Z) (N′-(2-hydroxy-3-(piperidin-1-yl)propoxy)-3-pyridine-carboximidamide) with good water solubility (28 mg/mL in deionized water) and low molecular weight (351.272 g/mol) [34]. BGP-15 is a promising anti-diabetic drug. In a randomized double-blind, placebo-controlled study, BGP-15 has improved insulin sensitivity in insulin-resistant patients [35] as well as in rabbits and rats [36]. Moreover, BGP-15 protects against diabetic cardiomyopathy [37], and diabetic retinopathy [38], which are frequent complications of diabetes.

As the prevalence of vascular calcification and diabetes is continually increasing, new therapeutic approaches are urgently needed to reduce both the economic and public health burden of these diseases. In addition, given that BGP-15 is a promising anti-diabetic drug candidate protecting against diabetic complications, such as cardiomyopathy and retinopathy, it is vital to clarify its potential effect on vascular calcification, which is a common complication of diabetes.

The present study was aimed to investigate the potential of BGP-15, a promising novel anti-diabetic drug candidate, to prevent in vitro mineralization and osteochondrogenic transformation of VSMCs induced by high Pi in normal glucose (NG) condition. In addition, we examine its anti-calcific potential in high Pi-induced mineralization aggravated by the high glucose (HG) condition. Here, we demonstrate that BGP-15 dose-dependently attenuates both high Pi- and high Pi + HG-induced VSMC transformation and mineralization suggesting its therapeutic potential in diabetic and non-diabetic vascular calcification.

## 2. Results

### 2.1. HG Aggravates High Pi-Induced ECM Calcium Deposition and Pi Uptake of VSMCs

**Figure 1:**
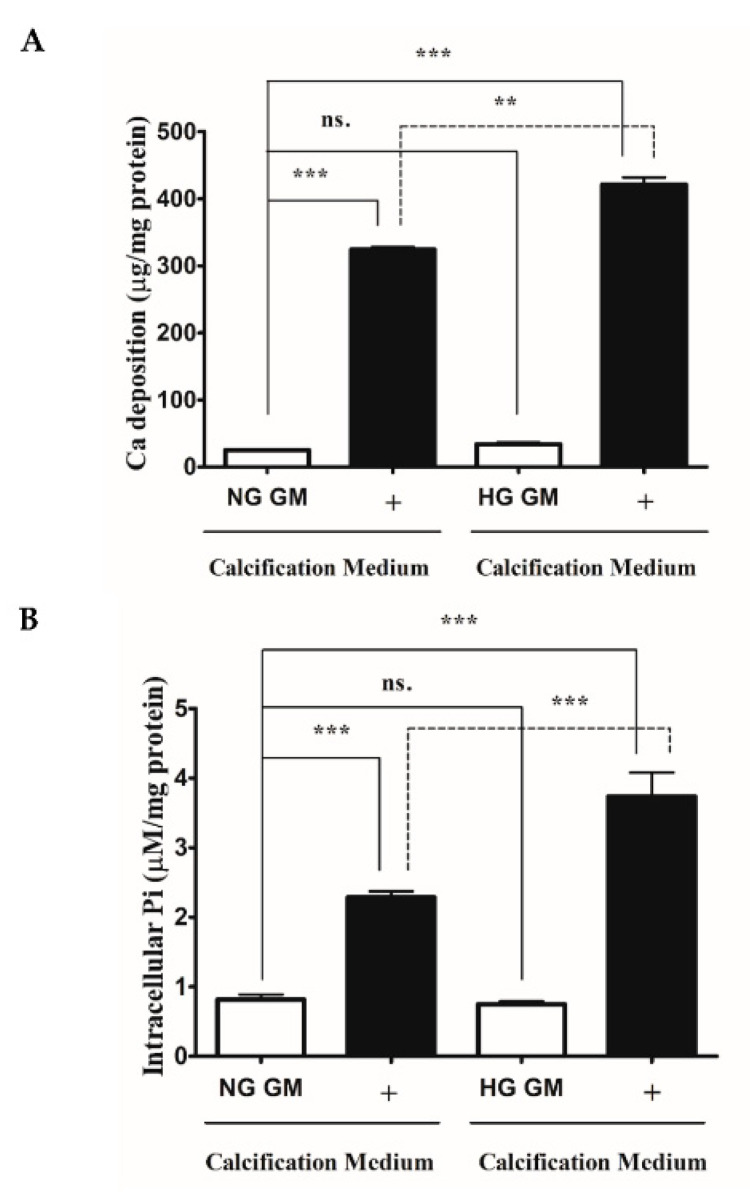
High glucose aggravates Pi-induced ECM calcium deposition and Pi uptake of VSMCs. VSMCs were cultured in calcification medium containing 3 mmol/L inorganic Pi under normal glucose (5.5 mM glucose) and high glucose (11 mM) conditions for 10 days. (**A**) Calcium content of solubilized ECM is presented. Data are expressed as mean ± SEM of three independent experiments. (**B**) Intracellular Pi levels are presented as mean ± SEM of three independent experiments. Statistical analysis was performed by the one-way ANOVA test followed by Bonferroni correction. A value of *p* < 0.05 was considered significant. ns: Non-significant; ** *p* < 0.01, *** *p* < 0.001.

HG and DM are known to enhance VSMCs calcification [3,22]. First, we analyzed whether the HG condition boosts ECM calcium deposition and Pi uptake of VSMCs under our experimental conditions. We showed that high Pi increased ECM calcium deposition (Figure 1A) and Pi uptake of VSMCs (Figure 1B), which was significantly aggravated by HG (Figure 1A,B). Our results corroborated earlier findings that HG aggravates VSMCs calcification in response to high Pi.

### 2.2. BGP-15 Mitigates High Pi-Induced ECM Calcium Deposition and Pi Uptake of VSMCs in Both Normal Glucose (NG) and High Glucose (HG) Conditions

**Figure 2:**
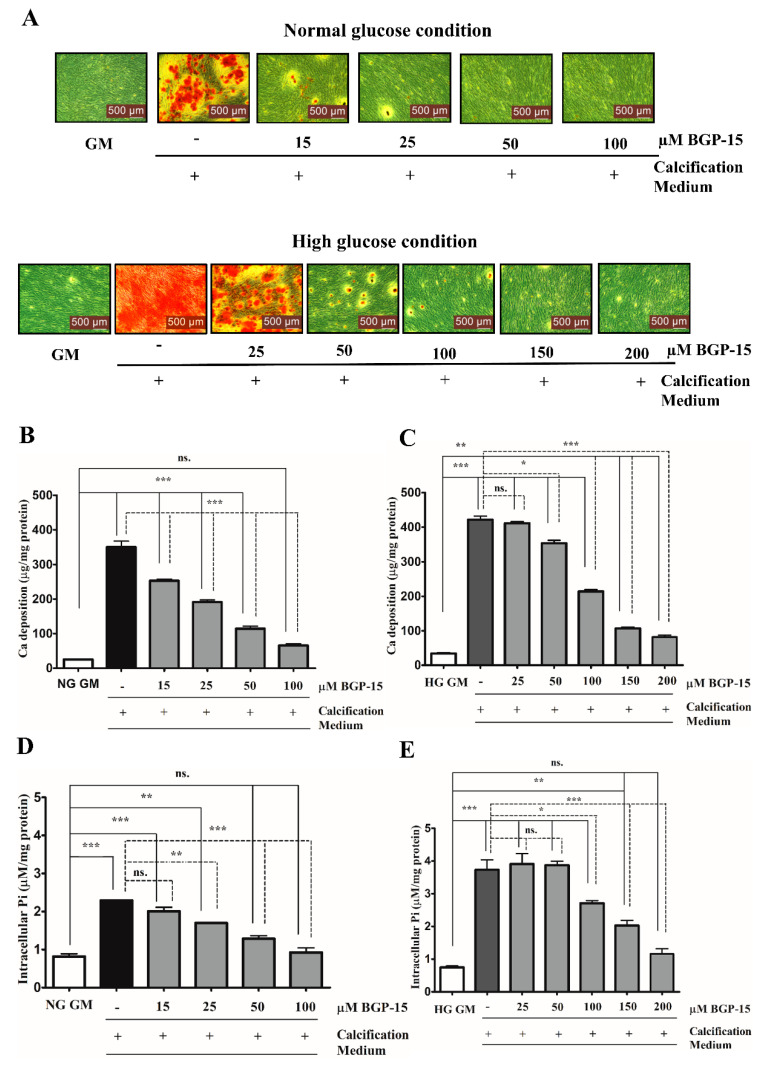
BGP-15 mitigates high Pi-induced ECM calcium deposition and Pi uptake of VSMCs in NG and HG conditions. VSMCs were cultured in calcification medium containing 3 mmol/L inorganic Pi under normal glucose (5.5 mM glucose; NG) and high glucose (11 mM; HG) conditions in the presence or absence of BGP-15 (15–100 µM BGP-15 in NG and 25–200 µM in HG condition) for 10 days. (**A**) Representative Alizarin Red staining is shown from three independent experiments. (**B**) Calcium content of solubilized ECM in NG and (**C**) HG conditions is presented. Data are expressed as mean ± SEM of three independent experiments. (**D**) Intracellular Pi levels in NG and (**E**) HG conditions are presented as mean ± SEM of three independent experiments. Statistical analysis was performed by the one-way ANOVA test followed by Bonferroni correction. A value of *p* < 0.05 was considered significant. ns: Non-significant; * *p* < 0.05, ** *p* < 0.01, *** *p* < 0.001.

To examine whether BGP-15 inhibits high Pi-induced mineralization of VSMCs under NG and HG conditions, VSMCs were cultured in growth medium (GM) or in calcification medium containing 3 mmol/L inorganic Pi under NG (5.5 mM glucose) and HG (11 mM glucose) conditions in the presence or absence of different concentrations of BGP-15 (15–100 µM BGP-15 in NG condition and 50–200 µM in HG condition) for 10 days (Figure 2A–E).

Calcium deposition by VSMCs was examined by Alizarin Red Staining, which revealed that Pi significantly increased mineral deposition compared to the untreated cells (GM) in NG (Figure 2A, upper panel) and HG conditions (Figure 2A, lower panel). We showed that BGP-15 significantly inhibited mineral deposition in a dose-dependent manner (Figure 2A).

To quantify ECM mineralization, we measured the calcium content of the ECM. We found that Pi alone increased the ECM calcium content (Figure 2B), which was aggravated by the HG condition (Figure 2C). Importantly, BGP-15 dose-dependently lowered calcium deposition in NG and HG conditions (Figure 2B,C).

The active uptake of Pi into the cells via the type III sodium-dependent Pi cotransporter Pit-1, has a crucial role in Pi-induced calcification of VSMCs [39]. The elevated Pi level is an independent risk factor for VC [9]. Therefore, we quantified the Pi uptake by measuring the intracellular Pi level in VSMCs. We showed that Pi markedly increased the intracellular Pi level (Figure 2D), which was aggravated by the HG condition (Figure 2E). BGP-15 dose-dependently lowered the Pi uptake in both conditions (Figure 2D,E). Importantly, BGP-15 showed no toxic effect on VSMCs (Supplementary Figure S1A,B). As 25 µM of BGP-15 did not affect calcification, we omitted this concentration from further experiments. Overall, these data demonstrated that BGP-15 effectively inhibited Pi-induced mineral deposition and Pi uptake by human VSMCs in a dose-dependent manner in both conditions.

### 2.3. BGP-15 Inhibits High Pi-Induced Loss of Smooth Muscle Cell-Specific Markers in Both NG and HG Conditions

**Figure 3:**
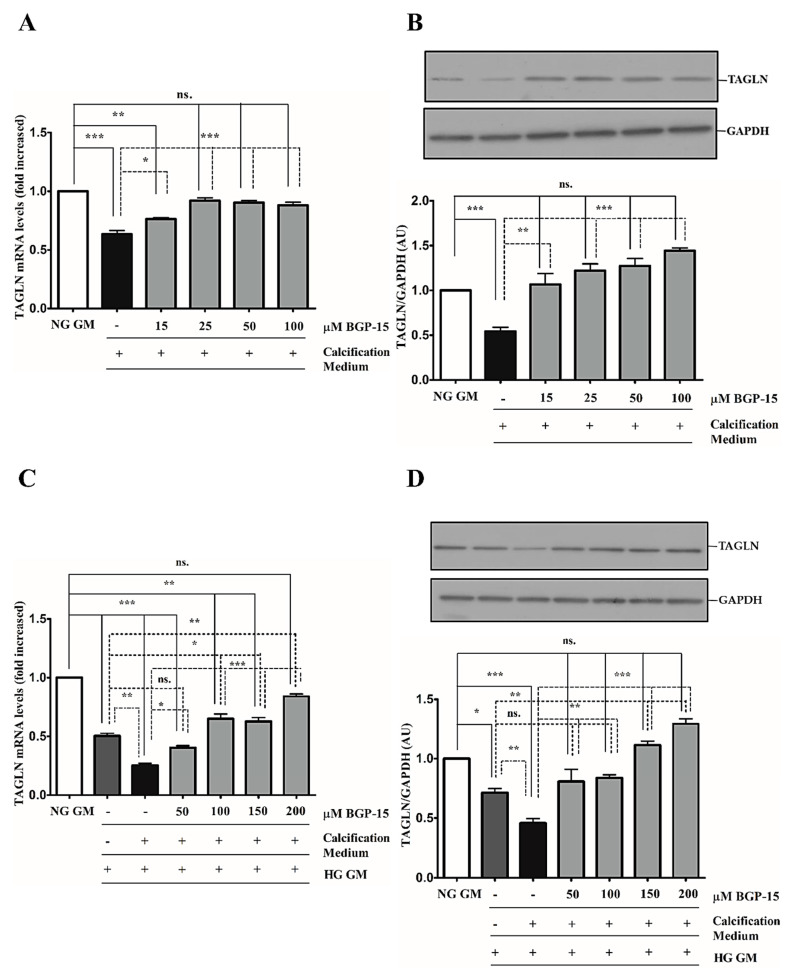
BGP-15 counteracts the loss of TAGLN induced by high Pi in NG and HG conditions. VSMCs were cultured in calcification medium containing 3 mmol/L inorganic Pi under NG (5.5 mM glucose) and HG (11 mM) conditions in the presence or absence of BGP-15 (15–100 µM BGP-15 in NG and 50–200 µM in HG conditions) for 10 days. Expression of TAGLN was analyzed by RT-qPCR and immunoblot (A-D). Relative expression of TAGLN mRNA (**A**) and protein (**B**) in NG and HG ((**C**): mRNA, (**D**): protein) is presented as mean ± SEM of three independent experiments. Relative mRNA expression was normalized to RNA45S5, while protein expression to GAPDH. Statistical analysis was performed by the one-way ANOVA test followed by Bonferroni correction. A value of *p* < 0.05 was considered significant. * *p* < 0.05, ** *p* < 0.01, *** *p* < 0.001.

Downregulation of smooth muscle-specific markers, such as transgelin (TAGLN) and smooth muscle α-actin (ACTA-2), is a common feature of VSMC calcification and osteochondrogenic phenotypic transition [28]. Therefore, we next examined whether BGP-15 could counteract Pi-induced loss of smooth muscle-specific markers. We showed that high Pi downregulated TAGLN expression (Figure 3A,B), which was further decreased by the HG condition (Figure 3C,D). Importantly, BGP-15 counteracted Pi-induced loss of TAGLN in both conditions (Figure 3A–D).

**Figure 4:**
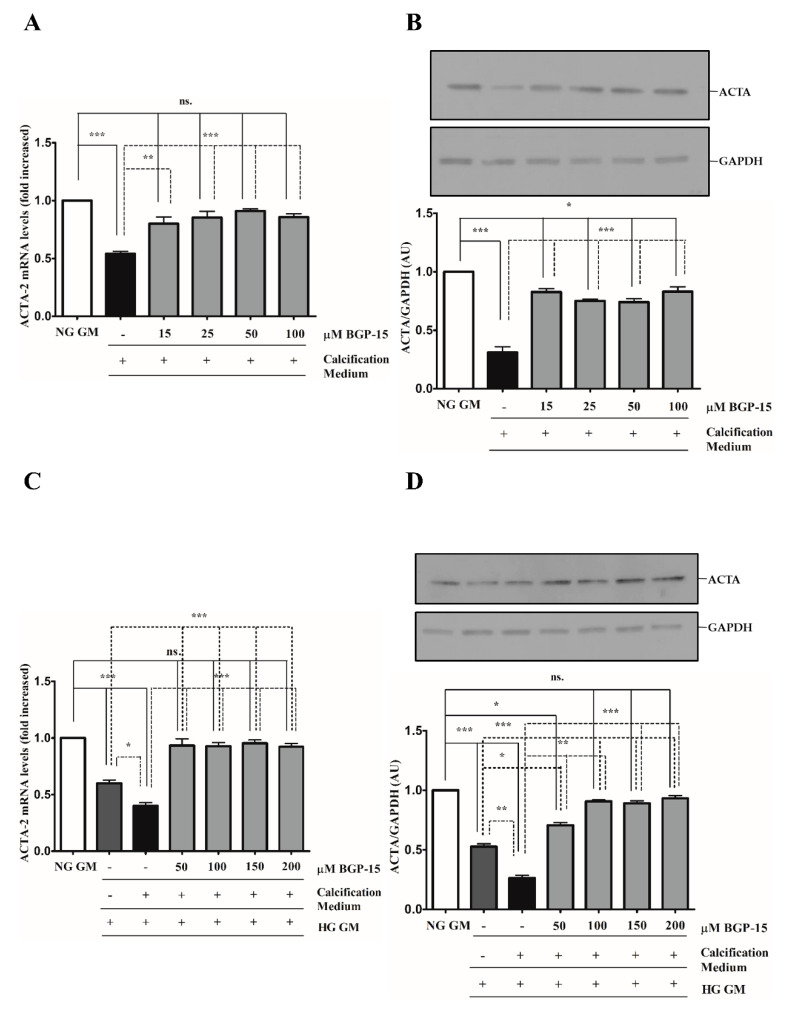
BGP-15 counteracts the loss of ACTA-2 induced by high Pi in NG and HG conditions. VSMCs were treated as in Figure 2. Expression of ACTA-2 was analyzed by RT-qPCR and immunoblot (**A**–**D**). Relative expression of ACTA-2 mRNA (**A**) and protein (**B**) in NG and HG ((**C**): mRNA; (**D**): protein) conditions are shown as mean ± SEM of three independent experiments. Relative mRNA expression was normalized to RNA45S5, while protein expression to GAPDH. Statistical analysis was performed by the one-way ANOVA test followed by Bonferroni correction. A value of *p* < 0.05 was considered significant. * *p* < 0.05, ** *p* < 0.01, *** *p* < 0.001.

We showed that high Pi downregulated ACTA-2 expression (Figure 4A,B), which was further decreased by the HG condition (Figure 4C,D). Similar to TAGLN, the HG condition decreased ACTA-2 level compared to NG independently of high Pi (Figure 4C,D). In line with TAGLN, BGP-15 restored ACTA-2 expression in NG and HG conditions (Figure 4A–D).

Altogether, HG aggravated high Pi-induced loss of VSMC-specific markers, which was counteracted by BGP-15.

### 2.4. BGP-15 Inhibited Osteochondrogenic Gene Expression Induced by High Pi in Both NG and HG Conditions

**Figure 5:**
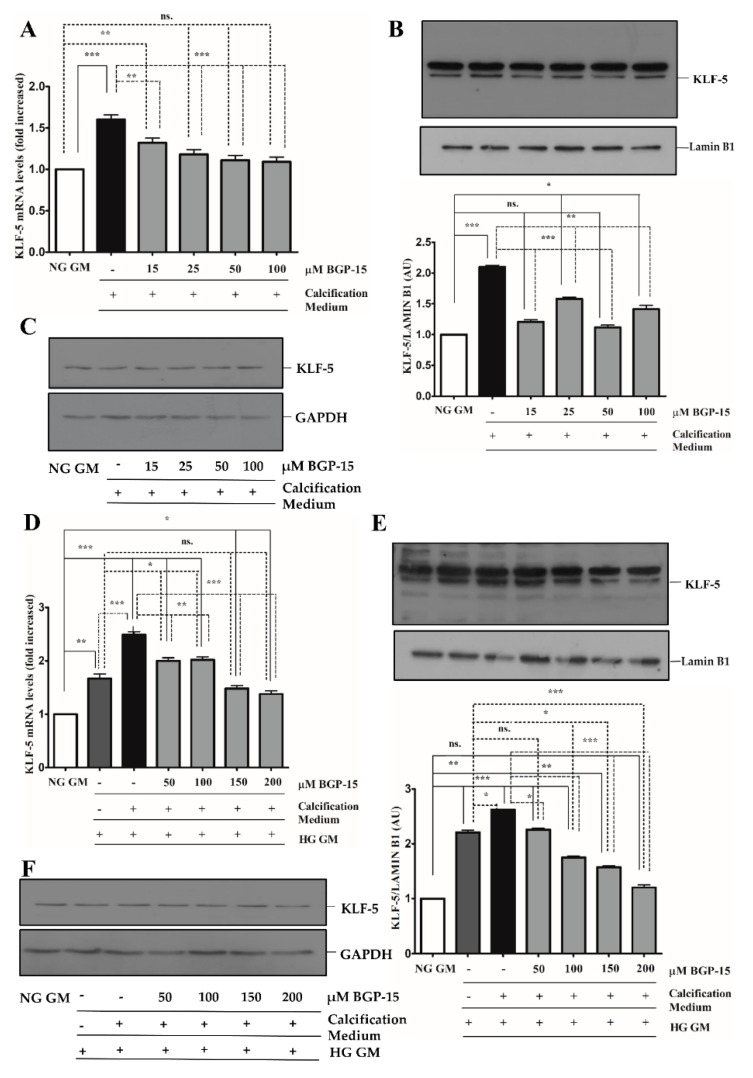
BGP-15 decreases KLF-5 expression and nuclear translocation induced by high Pi in NG and HG conditions. VSMCs were treated as in Figure 2. Expression of KLF-5 was analyzed by RT-qPCR and immunoblot (**A**–**F**). Relative expression of KLF-5 mRNA (**A**) and protein (**B**,**C**) in NG condition ((**B**): Protein from nuclear extracts normalized to Lamin B1; (**C**): Cytosolic fractions normalized to GAPDH). Relative expression of KLF-5 mRNA (**D**) and protein (**E**,**F**) in HG condition ((**E**): Protein from nuclear extracts normalized to Lamin B1; (**F**): Cytosolic fractions normalized to GAPDH). Data are presented as mean ± SEM of three independent experiments. Relative mRNA expression was normalized to RNA45S5. Statistical analysis was performed by the one-way ANOVA test followed by Bonferroni correction. A value of *p* < 0.05 was considered significant. * *p* < 0.05, ** *p* < 0.01, *** *p* < 0.001.

**Figure 6:**
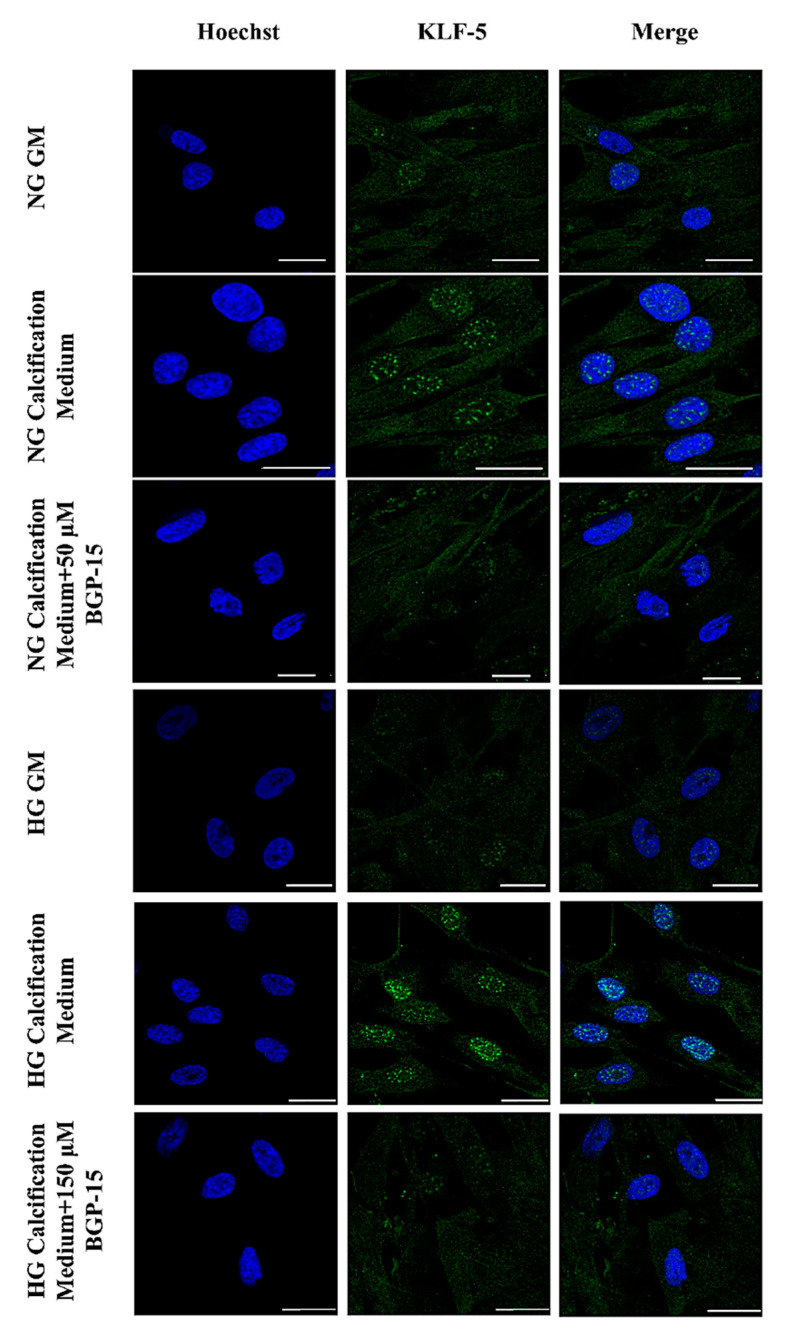
BGP-15 decreases nuclear translocation of KLF-5 induced by high Pi in NG and HG conditions. VSMCs were treated as in Figure 2 using 50 µM BGP-15 under NG and 150 µM under HG conditions for 10 days, then plated to glass coverslips. Nuclear translocation of KLF-5 was analyzed by immunofluorescence. Nuclei were counterstanied with Hoechst staining. Bar represents 25 µm.

**Figure 7:**
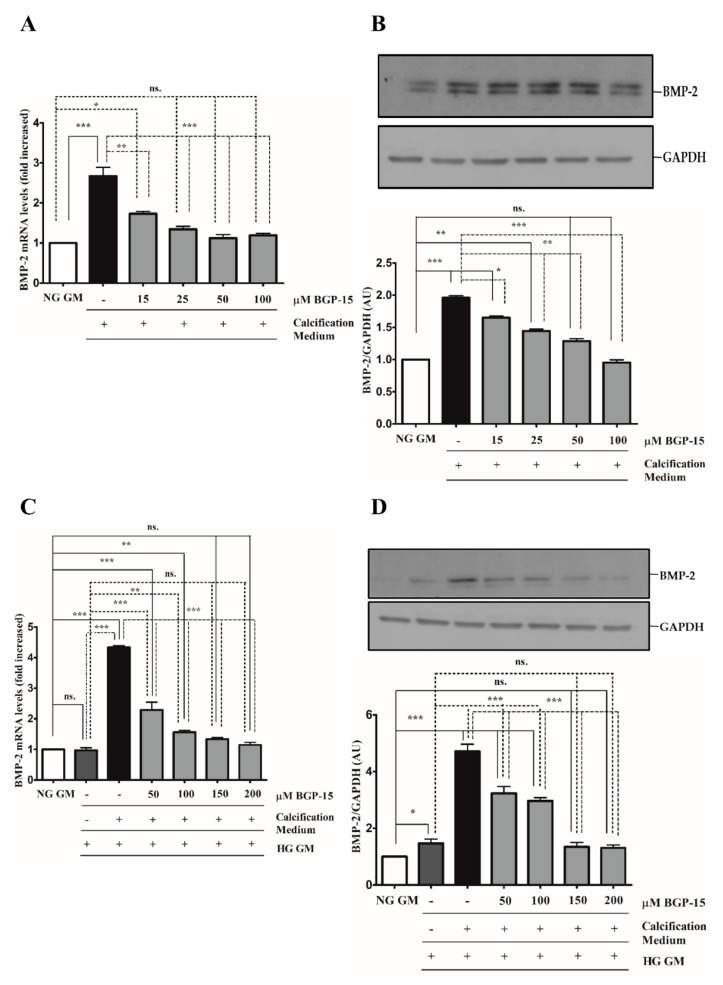
BGP-15 decreases BMP-2 expression induced by high Pi in NG and HG conditions. VSMCs were treated as in Figure 2. Expression of BMP-2 was analyzed by RT-qPCR and immunoblot (**A**–**D**). Relative expression of BMP-2 mRNA (**A**) and protein (**B**) in both NG and HG conditions ((**C**): mRNA; (**D**): protein) is shown as mean ± SEM of three independent experiments. Relative mRNA expression was normalized to RNA45S5, while protein expression to GAPDH. Statistical analysis was performed by the one-way ANOVA test followed by Bonferroni correction. A value of *p* < 0.05 was considered significant. * *p* < 0.05, ** *p* < 0.01, *** *p* < 0.001.

**Figure 8:**
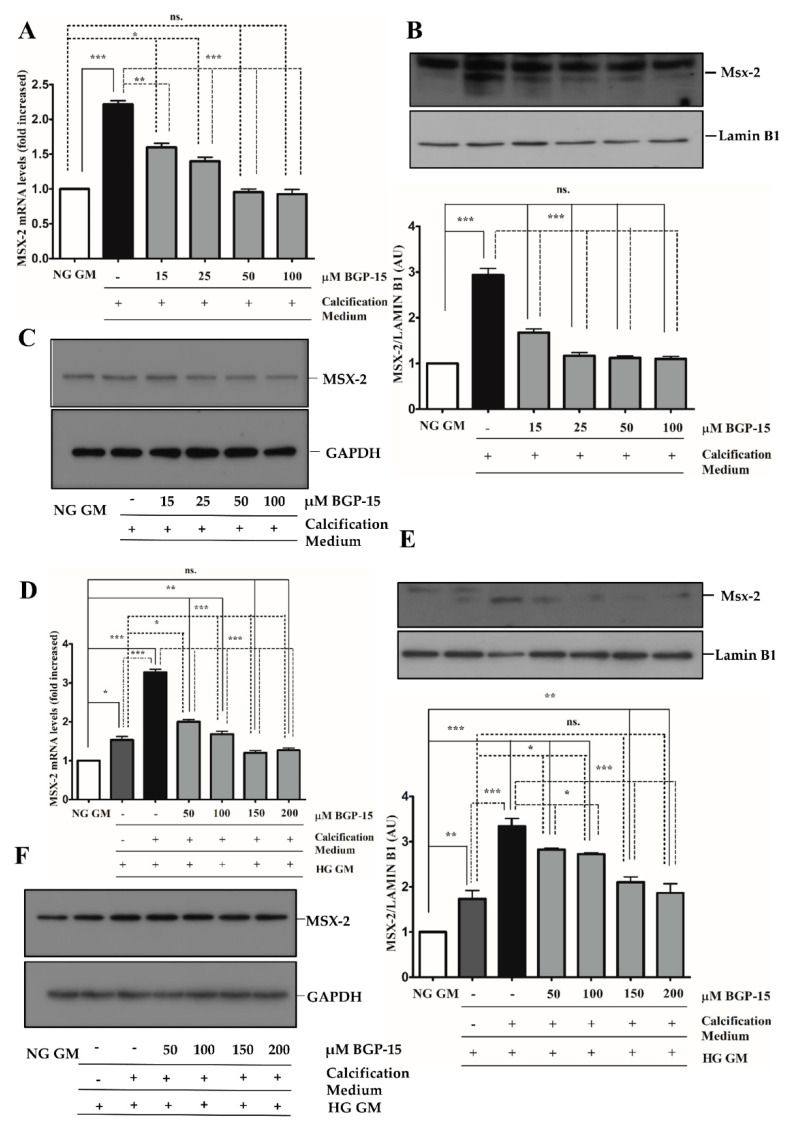
BGP-15 decreases Msx-2 expression and nuclear translocation induced by high Pi in NG and HG conditions. VSMCs were treated as in Figure 2. Expression of Msx-2 was analyzed by RT-qPCR and immunoblot (**A**–**F**). Expression of Msx-2 was analyzed by RT-qPCR and immunoblot (**A**–**F**). Relative expression of Msx-2 mRNA (**A**) and protein (**B**,**C**) in NG condition ((**B**): Protein from nuclear extracts normalized to Lamin B1; (**C**): Cytosolic fractions normalized to GAPDH). Relative expression of Msx-2 mRNA (**D**) and protein (**E**,**F**) in HG condition ((**E**): Protein from nuclear extracts normalized to Lamin B1, (**F**): Cytosolic fractions normalized to GAPDH). Data are presented as mean ± SEM of three independent experiments. Relative mRNA expression was normalized to RNA45S. Statistical analysis was performed by the one-way ANOVA test followed by Bonferroni correction. A value of *p* < 0.05 was considered significant. * *p* < 0.05, ** *p* < 0.01, *** *p* < 0.001.

**Figure 9:**
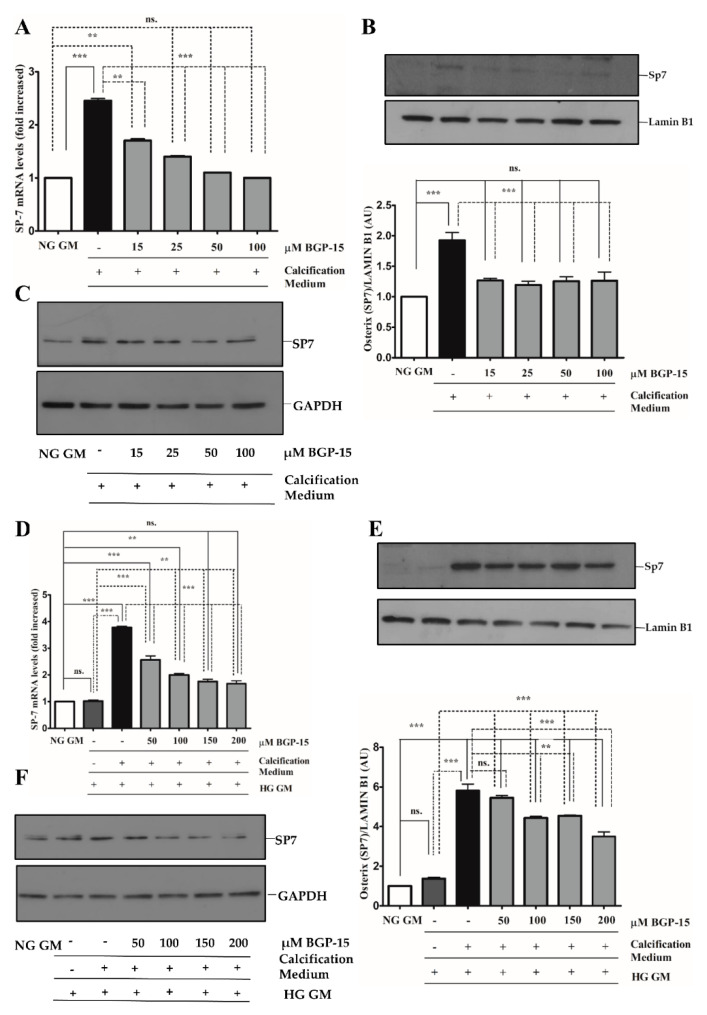
BGP-15 decreases Sp7 expression and nuclear translocation induced by high Pi in NG and HG conditions. VSMCs were treated as in Figure 2. Expression of Sp7 were analyzed by RT-qPCR and immunoblot (**A**–**D**). Expression of Sp7 was analyzed by RT-qPCR and immunoblot (**A**–**F**). Relative expression of Sp7 mRNA (**A**) and protein (**B**,**C**) in NG condition ((**B**): Protein from nuclear extracts normalized to Lamin B1; (**C**): Cytosolic fractions normalized to GAPDH). Relative expression of Sp7 mRNA (**D**) and protein (**E**,**F**) in HG condition ((**E**): Protein from nuclear extracts normalized to Lamin B1; (**F**): Cytosolic fractions normalized to GAPDH). Data are presented as mean ± SEM of three independent experiments. Relative mRNA expression was normalized to RNA45S. Statistical analysis was performed by the one-way ANOVA test followed by Bonferroni correction. A value of *p* < 0.05 was considered significant. ** *p* < 0.01, *** *p* < 0.001.

VSMCs phenotypic switch toward an osteochondrogenic phenotype involves the expression of osteoblast-specific markers. To decipher the molecular mechanism of how BGP-15 inhibited VSMCs mineralization, we analyzed the expression of several key markers of osteochondrogenic transformation, which are KLF-5, Msx-2, BMP-2, and Sp7 (Figure 5, Figure 6, Figure 7, Figure 8 and Figure 9).

The KLF-5 transcription factor plays a critical role in VSMCs osteochondrogenic transformation and mineralization [27]. We showed that high Pi significantly induced KLF-5 mRNA expression (Figure 5A) and KLF-5 protein nuclear translocation in NG condition (Figure 5B,C), which were dose-dependently inhibited by BGP-15.

Compared to the NG condition, HG aggravated high Pi-induced expression and nuclear translocation of KLF-5, which was significantly decreased by BGP-15 (Figure 5D–F). To corroborate this, we next analyzed the KLF-5 nuclear translocation by immunofluorescence. We showed that high Pi facilitated nuclear translocation of KLF-5, which was aggravated by HG (Figure 6). Importantly, BGP-15 inhibited high Pi-induced nuclear translocation of KLF-5 in both NG and HG conditions (Figure 6).

The BMP-2/Msx-2 axis has a key role in VC. Therefore, we first analyzed whether BGP-15 inhibits BMP-2 in response to high Pi under NG and HG conditions. We showed that high Pi induced BMP-2 expression (Figure 7A,B), which was aggravated by HG (Figure 7C,D) both of which were significantly reduced by BGP-15 in a dose-dependent manner.

Additionally, we showed that BGP-15 lowered high Pi-induced expression and nuclear translocation of Msx-2 not only in the NG condition (Figure 8A–C), but it also reduced high Pi-induced expression boosted by HG (Figure 8D–F). Importantly, HG also significantly increased Msx-2 expression and nuclear translocation when high Pi was absent.

The Sp7 (Osterix) transcription factor was also an important player in osteoblast differentiation and bone formation [30]. Therefore, we next examined whether BGP-15 also inhibits Sp7 expression and its nuclear translocation. We showed that high Pi induced Sp7 mRNA expression (Figure 9A) and nuclear translocation of Sp7 protein (Figure 9B,C), which were aggravated by the HG condition (Figure 9D–F). Importantly, both Pi and Pi + high glucose-triggered Sp7 expression were blunted by BGP-15 in a dose-dependent manner.

On the whole, high Pi-induced osteochondrogenic gene expressions in VSMCs were significantly boosted when HG was present. Importantly, the BGP-15 dose-dependently prevented KLF-5, BMP-2/Msx-2, and Sp7 expressions together with nuclear translocation of KLF-5, Msx-2, and Sp7. Our results also demonstrated that the HG condition alone predisposed VSMCs to osteochondrogenic phenotypic switch.

### 2.5. BGP-15 Inhibited PDK-4 Expression Induced by High Pi in Both NG and HG Conditions during the Osteochondrogenic Transformation of VSMCs to Osteoblast-Like Cells

**Figure 10:**
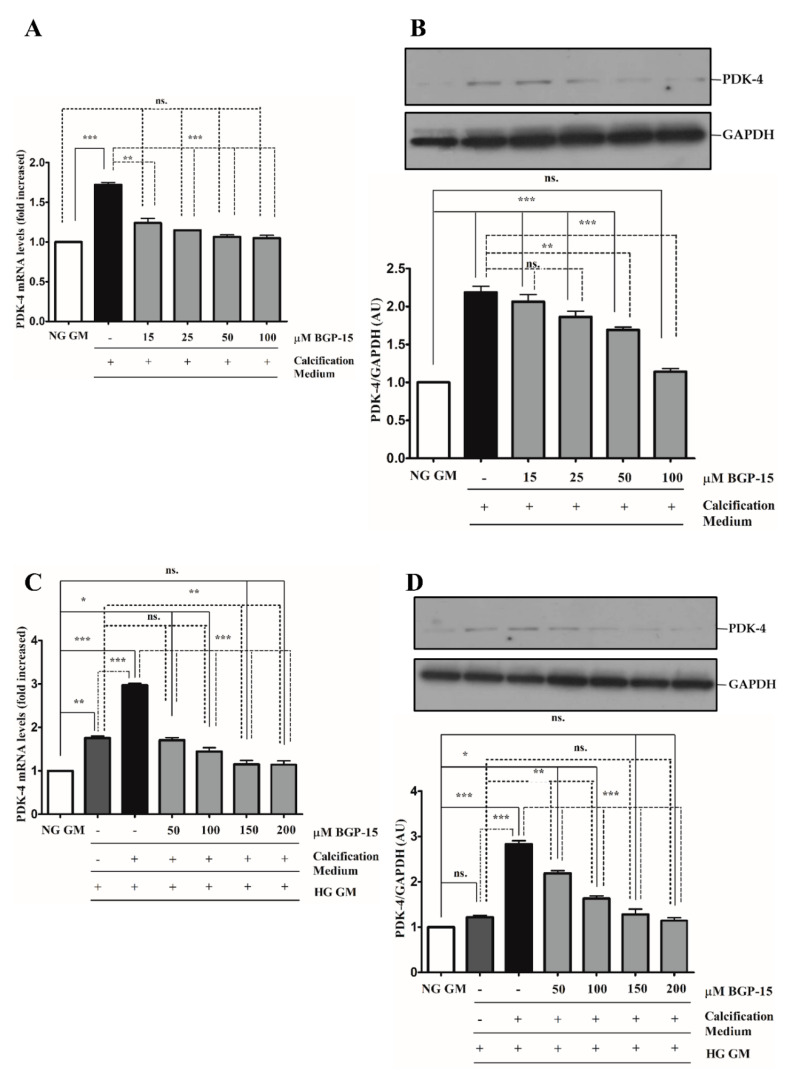
BGP-15 decreases PDK-4 expression induced by high Pi in NG and HG conditions. VSMCs were treated as in Figure 2. Expression of PDK-4 was analyzed by RT-qPCR and immunoblot (**A**–**D**). Relative expression of PDK-4 mRNA (**A**) and protein (**B**) in NG and HG ((**C**): mRNA; (**D**): protein) conditions is presented as mean ± SEM of three independent experiments. Relative mRNA expression was normalized to RNA45S, while protein expression to GAPDH. Statistical analysis was performed by the one-way ANOVA test followed by Bonferroni correction. A value of *p* < 0.05 was considered significant. * *p* < 0.05, ** *p* < 0.01, *** *p* < 0.001.

Pyruvate dehydrogenase kinase-4 (PDK-4) has an important role in vascular calcification [32]. Next, we explored whether BGP-15 inhibits PDK-4 induction in response to high Pi in both NG and HG conditions. We showed that high Pi induced PDK-4 expression (Figure 10A,B), which was markedly boosted by HG (Figure 10C,D). Importantly, BGP-15 inhibited PDK-4 expression in both conditions (Figure 10A–D). Interestingly, HG also slightly elevated PDK-4 expression even when high Pi was absent (Figure 10C,D).

**Figure 11:**
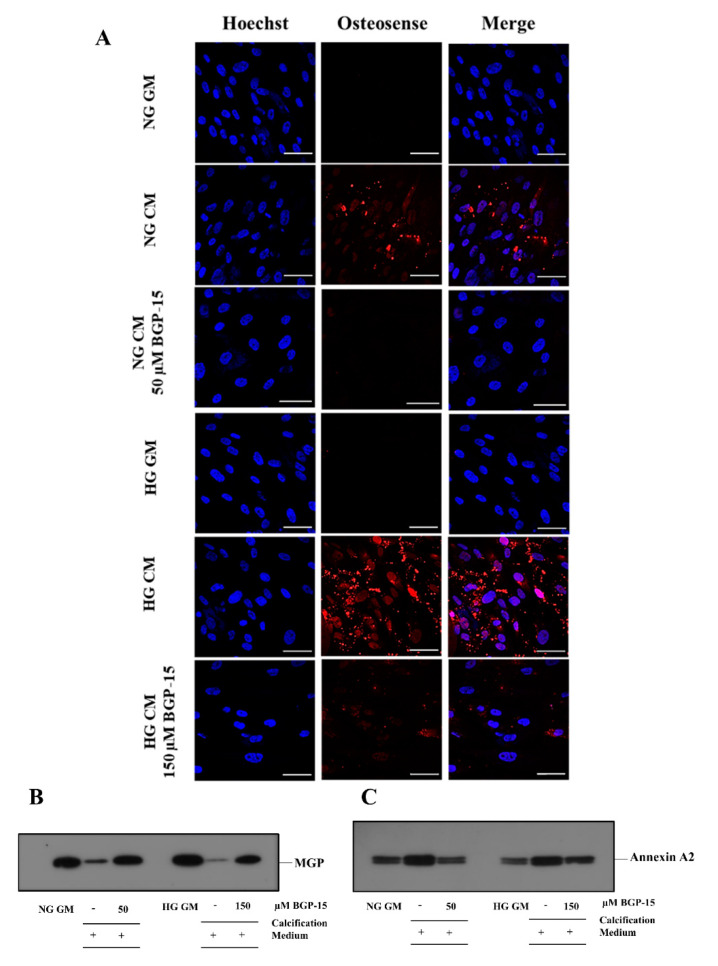
BGP-15 decreases microcalcification, the loss of MGP, and the induction of Annexin A2 induced by high Pi in NG and HG conditions. (**A**) VSMCs were treated as in Figure 2 using 50 µM BGP-15 under NG and 150 µM under HG conditions for 10 days, then plated to glass coverslips. Calcium deposition was visualized by OsteoSense 680. Nuclei were counterstanied with Hoechst staining. Bar represents 50 µm. (**B**) BGP-15 attenuates the loss of MGP induced by high Pi in NG and HG conditions. VSMCs were treated as in Figure 2 using 50 µM BGP-15 under NG and 150 µM under HG conditions for 10 days, EVs were analyzed by immunoblotting. (**C**) BGP-15 decreases Annexin A2 expression induced by high Pi in NG and HG conditions. VSMCs were treated as in Figure 2 using 50 µM BGP-15 under NG and 150 µM under HG conditions for 10 days, EVs were analyzed by immunoblotting.

Elevated extracellular calcium in VSMC cultures was also visualized by confocal microscopy using the near-infrared-based bisphosphonate calcium tracer OsteoSense680. We showed that high Pi induced calcium deposition in VSMCs, which was aggravated by HG (Figure 11A). BGP-15 (50 µmol/L in NG and 150 µmol/L in HG conditions) significantly attenuated high Pi-induced calcification in VSMC cultures in both NG and HG conditions (Figure 11A).

Increased extracellular calcium facilitates the secretion of calcifying extracellular vesicles (EVs) from VSMCs [13], which play an important role in VC [14]. Therefore, we examined the presence of calcification inhibitor Matrix Gla protein (MGP) and calcification promoter Annexin A2 in isolated EVs. We showed that the MGP load decreased, whereas Annexin A2 increased in response to high Pi in both conditions, which were counteracted by BGP-15 (Figure 11B,C).

## 3. Discussion

In this study, we evaluated the effect of BGP-15, an emerging anti-diabetic drug candidate, on osteochondrogenic transformation and mineralization of VSMCs. We showed for the first time that BGP-15 protected against high Pi-induced osteogenic trans-differentiation and mineralization of VSMCs in both NG and HG conditions.

The prevalence of vascular diseases and diabetes mellitus are continually rising worldwide. Vascular calcification is a common, life-threatening complication of diabetes with significant mortality and morbidity [40,41] that necessitates exploring possible remedies [42,43,44]. Given the strong correlation between diabetes and vascular calcification, exploring potential drug candidates that simultaneously treat both hyperglycemia and vascular calcification has high clinical relevance. BGP-15 is a well tolerable drug candidate with remarkable anti-diabetic properties [35,36]. Despite its known anti-diabetic activity, the effects of BGP-15 on diabetic vascular complications have not been yet explored.

Previous studies have shown that HG promotes VSMCs mineralization [21,22]. Along lines with these studies, we showed that HG strongly aggravated the osteogenic trans-differentiation and mineralization of VSMCs in response to high Pi.

Alterations in the biological characteristics of VSMCs are major determinants of vascular calcification. In their contractile phenotype, VSMCs are characterized by high expressions of smooth muscle-specific markers, among them ACTA and TAGLN. In response to environmental stimuli such as high Pi, VSMCs undergo a phenotypic switch into an osteoblast-like phenotype, which is characterized by the downregulation of smooth muscle-specific markers being a common feature of VSMCs calcification [28]. Evidence shows that diabetes and HG predispose VSMCs to phenotypic switch. In diabetic rat carotid arteries, expression of smooth muscle-specific markers including ACTA, myosin heavy chain, and calponin-1 is significantly decreased [45]. Others also have found that contractile smooth muscle-specific markers are decreased in response to hyperglycemia [46]. Along the lines with these results, we showed that both high Pi and HG triggered the loss of SMC-specific markers, and the HG condition aggravated high Pi-induced downregulation of SMC markers. Here, we found that BGP-15 attenuated the loss of SMC-specific markers triggered by high Pi in both NG and HG conditions. These results indicate that BGP-15 may help prevent the downregulation of SMC-specific markers, which potentially predisposes VSMCs to phenotypic switch.

Osteochondrogenic phenotypic switch and mineralization of VSMCs are governed by the activation of osteogenic proteins such as KLF-5, BMP-2, Msx-2, and Sp7. KLF-5 has a critical role in the initiation of high Pi-induced VSMC calcification and its expression is increased in the calcified aorta of adenine-induced uremic rats [27]. In the murine model of atherosclerosis, KLF-5 expression is elevated, which is associated with atherosclerosis progression [47] supporting the role of KLF-5 in promoting vascular diseases. In the present study, we showed that high Pi induced KLF-5 expression and nuclear translocation both of which were significantly aggravated by HG. Importantly, BGP-15 attenuated both Pi and Pi + high glucose-induced KLF-5 expression and nuclear translocation suggesting the therapeutic potential of BGP-15 to ameliorate VSMCs phenotypic switch early at its initiation step.

The BMP-2/Msx-2 axis has a key role in vascular calcification. Msx-2 promotes vascular calcification [30] and enhances vascular mineralization in diabetic atherosclerotic calcification by governing osteochondrogenic reprogramming [48]. Msx-2 signaling is also involved in aortic calcification in diabetic nephropathy rat model [49]. HG increases the expression of BMP-2 and enhances the calcification of VSMCs [22], while elevated plasma BMP-2 levels are associated with atherosclerosis burden and coronary mineralization in type 2 diabetes [50]. These indicate a more potent activation of osteogenic reprogramming of VSMCs in diabetic conditions in a BMP-2/Msx-2 axis-mediated manner suggesting that inhibitors of BMP-2/Msx-2 activation might have a therapeutic benefit in vascular calcification. Here, we showed that BGP-15 effectively inhibited high Pi-induced BMP-2/Msx-2 expressions in both NG and HG conditions indicating that BGP-15 can be a valuable therapeutics to mitigate vascular mineralization in diabetic, as well as non-diabetic conditions.

Sp7 (Osterix) is also an important factor in osteoblast differentiation and bone formation [29]. This is supported by the fact that silencing of Sp7 expression inhibits VSMCs calcification in mice [51]. Here, we demonstrated that BGP-15 effectively inhibited both high Pi and high Pi-induced mineralization of VSMCs aggravated by HG supporting the potential role of BGP-15 to inhibit vascular calcification.

Our results showed that the HG condition induced the loss of smooth muscle-specific markers and slightly but not significantly triggered ostechondrogenic gene expressions in the absence of high Pi suggesting the role of HG to prime VSMCs for the osteochondrogenic phenotypic switch.

PDK-4 plays a key role in vascular calcification through autophagy inhibition and metabolic reprogramming, while inhibition of PDK-4 abrogates VSMCs calcification [32,33]. In addition, PDK-4 is upregulated in calcified vessels of atherosclerotic patients [32]. Multiple evidence shows that PDK-4 has an important role in the pathomechanism of metabolic diseases including insulin resistance and hyperglycemia [52,53,54]. Given that the PDK-4 inhibition is beneficial both in diabetes [53] and vascular calcification [33], it is reasonable to assume that lowering the PDK-4 activity might have a dual positive effect on diabetic vascular calcification. Our results published here supported this hypothesis since BGP-15 inhibited PDK-4 induction by high Pi in both NG and HG conditions.

Evidence shows that SMC-derived extracellular vesicles (EVs) play an important role in VC [14]. Matrix Gla protein (MGP) is a potent inhibitor of arterial calcification [55,56]. Homeostatic loading of EVs with calcification inhibitors such as MGP is disturbed during VSMCs mineralization [57,58]. Moreover, increased levels of calcification activators, among them Annexin A2, in extracellular vesicles are detected in response to calcification stimuli [59]. Here, we demonstrated that EVs released by calcifying VSMCs contained elevated Annexin A2 and decreased MGP loads, which were counteracted by BGP-15. This suggests that the inhibitory effect of BGP-15 in VC might be, at least partly, attributed to its effect attenuating calcifying vesicle release.

Over the past years, several anti-diabetic drugs have been reported to mitigate vascular calcification via different mechanisms. Metformin decreases high Pi-induced calcium deposition and inhibits the loss of smooth muscle-specific markers with the parallel inhibition of osteochondrogenic gene expression via cAMP-activated protein kinase [60]. Others have shown that metformin inhibits VSMCs calcification by inhibiting PDK-4 [61]. Another anti-diabetic drug, pioglitazone, attenuates VSMCs calcification by downregulating Wnt/β-catenin signaling [62]. These necessitate further research to explore the mechanism of action of BGP-15 in vascular calcification.

Our study has its strength and limitations. BGP-15 has not been investigated yet for mitigating vascular calcification. As a pilot study, in this work, we demonstrated that BGP-15 mitigated not only high Pi-induced osteochondrogenic transformation and mineralization of VSMCs, but also when it was dramatically aggravated by high glucose. However, animal studies will be needed to perform in the future to further support our in vitro findings.

Based on our results, we suggest that BGP-15 could be an effective therapeutic candidate against diabetes-associated vascular complications by preventing the loss of VSMC-specific markers, decreasing pro-calcific but increasing anti-calcific EV release, and inhibiting osteochondrogenic gene induction, together with the metabolic reprogramming of VSMCs induced by high Pi in NG and HG conditions.

## 4. Materials and Methods

### 4.1. Reagents

All the reagents were purchased from Sigma-Aldrich (St. Louis, MO, USA) unless otherwise stated.

### 4.2. Cell Culture

Human aortic smooth muscle cells (VSMCs) were purchased from Lonza (Allendale, NJ, USA Cat.: CC-2571; donor: Male, 22 years old, non smoking, non-diabetic, no heart disease). Cells were grown in Dulbecco’s Modified Eagle Medium (DMEM) containing 1000 mg/L (5.5 mmol/L) glucose supplemented with 5% fetal bovine serum (Life Technologies, Vienna, Austria), 100 U/mL penicillin, 100 μg/mL streptomycin, and neomycin referred to as the growth medium (GM). Cells were grown to confluence and used from passages 5 to 7. Media were changed every 2 days. To induce Pi-mediated calcification under normoglycemic conditions, GM was supplemented with inorganic phosphate (3 mmol/L referred to as a calcification medium) in the form of Na_2_HPO_4_/NaH_2_PO_4_ (pH 7.4) in the presence or absence of BGP-15 (15–100 μmol/L) for 10 days. According to our previous study [43], 10 days is optimal to study VSMCs calcification under our experimental condition. To induce Pi mediated calcification under hyperglycemic conditions, GM was supplemented with inorganic phosphate (3 mmol/L) in a form of Na_2_HPO_4_/NaH_2_PO_4_ (pH 7.4) and glucose (2000 mg/L final glucose concentration; 11 mmol/L) in the presence or absence of BGP-15 (50–200 μmol/L) for 10 days. Data are representative of three independent experiments.

### 4.3. MTT Assay

Cell viability was determined by the MTT assay. Briefly, cells were cultured and treated as described above in 96-well plates for the indicated time. Then, cells were washed with PBS, and 100 μL of 3-[4,5-dimethylthiazol-2-yl]-2,5-diphenyl-tetrazolium bromide (0.5 mg/mL) solution in Hank’s Balanced Salt Solution with Ca^2+^ and Mg^2+^ was added. After a 90-min incubation, the MTT solution was removed, formazan crystals were dissolved in 100 μL of DMSO and optical density was measured at 570 nm.

### 4.4. Alizarin Red Staining

Cells were rinsed with phosphate-buffered saline (PBS) pH 7.4 without Ca^2+^ and Mg^2+^ and fixed with 4% paraformaldehyde for 10 min at room temperature. Then, cells were washed with PBS without Ca^2+^ and Mg^2+^ and stained with 2% Alizarin Red S for 10 min. Excessive dye was removed by several washes in deionized water. Extracellular Ca^2+^ deposition was stained in red color.

### 4.5. Quantification of Calcium Deposition

Cells grown on 24-well plates were rinsed twice with PBS pH 7.4 without Ca^2+^ and Mg^2+^ and decalcified with 0.6 mol/L HCl for 30 min at 37 °C. After decalcification, cells were solubilized with a solution of NaOH 0.1 mol/L and SDS 0.1%, and the protein contents of samples were measured with the bicinchoninic acid (BCA) protein assay kit (Pierce, Rockford, IL, USA). The calcium content of the supernatants was determined by the QuantiChrom Calcium Assay Kit (Gentaur, Brussels, Belgium) and normalized to the protein content and expressed as μg/mg protein.

### 4.6. Measurement of Intracellular Inorganic Phosphate

The intracellular inorganic phosphate concentration was determined by colorimetric analysis using the QuantiChrom^TM^ Phosphate Assay Kit (Gentaur, Brussels, Belgium). Cells were rinsed twice with PBS pH 7.4 without Ca^2+^ and Mg^2+^ and then lysed with a solubilization buffer (1% Triton-X 100, 0.5% Igepal CA-630, 10% protease inhibitor, 150 mmol/L NaCl, 5 mmol/L EDTA, 10 mmol/L Tris). The intracellular Pi concentration of the cells was normalized to the protein content expressed as μmol/mg protein.

### 4.7. RNA Isolation and Quantitative Reverse Transcription–Polymerase Chain Reaction

Vascular smooth muscle cells were grown on 24-well plates and total RNA was isolated using TRIzol (Invitrogen, Carlsbad, CA, USA). The cDNA was synthesized using a High-Capacity cDNA Reverse Transcription Kit (Applied Biosystems, Foster City, CA, USA) for RT-PCR. Real-time polymerase chain reactions were performed using fluorescent TaqMan probes. TaqMan gene expression assays for BMP-2 (Hs00154192_m1), Msx-2 (Hs00741177_m1), PDK-4 (Hs01037712_m1), smooth muscle α-actin/ACTA2 (Hs00426895_g1, MYH11 (Hs00975796_m1), Sp7 (Hs01866874_s1), KLF-5 (Hs001561745_m1), and GAPDH (Hs02786624_g1) were purchased from Thermo Scientific, USA. Gene expressions were normalized to GAPDH. Polymerase chain reactions were carried out using the iCycler iQ Real-Time PCR system (Bio-Rad, Hercules, CA, USA), and the results represent the relative mRNA expression of target genes normalized RNA45S5 (Hs05627131_gH) mRNA levels.

### 4.8. Preparation of Whole-Cell Lysates, Cytosolic and Nuclear Fractions

To prepare whole cell lysates, VSMCs were washed with PBS pH 7.4 without Ca^2+^ and Mg^2+^ then lysed using a RIPA buffer (50 mmol/L Tris [pH 7.5], 150 mmol/L NaCl, 1% Igepal CA-630, 1% sodium deoxycholate, 0.1% SDS) containing 10% Complete Mini Protease Inhibitor Cocktail and 10% PhosSTOP phosphatase inhibitor cocktail, and incubated for 20 min on ice. Lysates were centrifuged at 14,000× *g*, 4 °C for 20 min. Supernatants were used as whole-cell extracts.

For cytosolic and nuclear fraction separation, cells were rinsed with PBS pH 7.4 without Ca^2+^ and Mg^2+^ then scraped with an ice-cold hypotonic buffer (20 mmol/L HEPES, 250 mmol/L Sucrose, 10 mmol/L KCl, 2 mmol/L MgCl_2_) containing 10% Complete Mini Protease Inhibitor Cocktail and 10% PhosSTOP phosphatase inhibitor cocktail on ice. Using 1 mL syringe, the cell suspension was passed through a 28 G needle 30 times then swelled on ice for 20 min. Lysates were centrifuged at 15,000× *g*, 4 °C for 15 min. Supernatants were collected as cytosolic fractions. This procedure was repeated four more times. Finally, the nuclear pellets were dissolved in RIPA buffer supplemented with 10% Complete Mini Protease Inhibitor Cocktail and 20% PhosSTOP phosphatase inhibitor cocktail and centrifuged at 16,000× *g*, 4 °C for 15 min. Supernatants were used as nuclear fractions. The protein content was determined using the bicinchoninic acid assay (Pierce BCA Protein Assay Kit, Thermo Fisher Scientific, Waltham, MA, USA).

### 4.9. Immunoblotting

Cell extracts (25 μg protein) were electrophoresed on 10% Tris–glycine SDS-gels and proteins were transferred to 0.45 μm nitrocellulose membrane (GE Healthcare, Chicago, IL, USA). Membranes were blocked with 5% *w*/*v* milk for 60 min at room temperature. After blocking, membranes were incubated with antibodies against TAGLN (Proteintech, Manchester, UK, Cat: 10493-1-AP), ACTA (Proteintech, Manchester, UK, Cat: 14395-1-AP), PDK-4 (Abcam, Cambridge, UK, Cat: ab88063), SP7/Osterix (Novus Biologicals, Centennial, CO, USA, Cat: MAB-7547), BMP-2 (Abcam, Cambridge, UK, Cat: AB14933), MSX-2 (Novus Biologicals, Centennial, CO, USA, Cat: NBP1-85445), KLF-5 (Genetex, Irvine, CA, USA, Cat: GTX103289), AnnexinA2 (Cell Signaling Technologies, Danvers, MA, USA, Cat: 8235), and MGP (Novus Biologicals, Bio-Techne Ltd., Abingdon, UK, Cat: NBP2-45844) at 4 °C overnight. Antigen-antibody complexes were visualized with the horseradish peroxidase chemiluminescence system (Advansta, Menlo Park, CA, USA). Protein bands were normalized to GAPDH (Proteintech, Manchester, UK, Cat: 60004-1-Ig) or Lamin B1 (Proteintech, Manchester, UK, Cat: 66095-I-Ig).

### 4.10. Confocal Microscopy

Cells were treated as described above in the presence or absence of 50 µmol/L (NG condition) and 150 µmol/L (HG condition) of BGP-15 for 10 days in 24-well plates. Cells were then trypsinized, plated to glass coverslips and further incubated for 16 h as in the growth medium or in calcification medium with or without BGP-15 as described above. Cells were then fixed with 4% paraformaldehyde, washed twice with phosphate buffered saline pH 7.4 (PBS), permeabilized with 0.5% Triton X-100, washed and blocked with 10% normal goat serum and 2% BSA for 60 min in PBS with 0.05% Tween-20 (PBS-T). Slides were then incubated overnight with KLF-5 antibody (Thermo Scientific, Waltham, MA, USA, Cat: 701885) at a dilution of 1:250 overnight at 4 °C in antibody dilution buffer (1% BSA in PBS-T), washed, then, further incubated with goat anti-rabbit IgG conjugated to Alexa Fluor 568 (Thermo Fisher Scientific, Waltham, MA, USA) at a dilution of 1:500 for 60 min. Nuclei were visualized with Hoechst. Samples were investigated with Lightning super-resolution microscopy using Leica Application Software X (Leica, Mannheim, Germany).

To analyze extracellular calcium deposition by confocal microscopy, cells were treated as described above trypsinized, plated to glass coverslips and further incubated for 16 h as in growth medium or in calcification medium with or without BGP-15 as described above in the presence of OsteoSense 680EX Fluorescent Imaging Agent (1:100), a near-infrared based bisphosphonate calcium tracer, for 24 h at 37 °C (PerkinElmer, Waltham, MA, USA). Cells were then fixed with 4% paraformaldehyde, washed twice with phosphate buffered saline pH 7.4 (PBS). Nuclei were visualized with Hoechst. Samples were investigated with Leica SP8 confocal microscope using Leica Application Software X (Leica, Mannheim, Germany).

### 4.11. Isolation of EVs

To examine the effect of BGP-15 on extracellular vesicle (EV) formation, we exposed VSMCs to 3 mmol/L inorganic Pi under NG and HG levels. To avoid a sharp decrease in serum concentration, fetal calf serum content of the experimental medium was gradually decreased from 5% to 0%. Therefore, VSMCs were cultured in calcification medium with normal and high glucose in the presence of 5% serum for 5 days, 3% for 3 days, and 1% for 2 days in the presence or absence of 50 µmol/L BGP-15 under normal glucose condition, while 150 µmol/L under high glucose condition. Then, 24 h before EV isolation, VSMCs were cultured under serum-free conditions. To limit the number of intact cells during EV preparations, cell culture supernatants were centrifuged at 400× *g* for 5 min at room temperature, then supernatants were transferred to new tubes and spinned again at 2000× *g* for 5 min at room temperature. Supernatants were then transferred to Amicon Ultra MWCO 3000 tubes and concentrated according to the manufacturer’s guide. Concentrated supernatants were diluted ten-fold with phosphate buffered saline pH 7.4 and concentrated again. The protein contents were measured with BCA assay as described earlier. The presence of Matrix Gla protein (MGP) and Annexin A2 in EVs was analyzed by immunoblotting.

### 4.12. Statistical Analysis

Data were analyzed by GraphPad Prism 5.02 software (GraphPad Software Inc., La Jolla, CA, USA). Statistical analysis was performed by the one-way ANOVA test followed by Bonferroni correction. A value of *p* < 0.05 was considered significant.

## Data Availability

The datasets generated during and/or analyzed during the current study are available from the corresponding author on reasonable request.

## Supplementary Materials

The following are available online at https://www.mdpi.com/article/10.3390/ijms22179263/s1.
