# Supplementary material for: BGP-15 Inhibits Hyperglycemia-Aggravated VSMC Calcification Induced by High Phosphate
Source: Int J Mol Sci. 2021 Aug 26;22(17):9263. doi: 10.3390/ijms22179263 (PMC8431374; doi:10.3390/ijms22179263)
Supplement: Supplementary file 1 [file ijms-22-09263-s001.zip › ijms-1327151-supplementary.pdf]

Article

BGP-15 Inhibits Hyperglycemia-Aggravated VSMC Calcification Induced by High Phosphate

Annamária Nagy [1,2#], Dávid Pethő [1,2#], Rudolf Gesztelyi [4], Béla Juhász [4], György Balla[3,5], Zoltán Szilvássy [4], József Balla [1,*], and Tamás Gáll [1,3]

[1] Division of Nephrology, Department of Medicine, Faculty of Medicine, University of Debrecen, 4032 Debrecen, Hungary;

[2] Kálmán Laki Doctoral School, Faculty of Medicine, University of Debrecen, 4032 Debrecen, Hungary

[3] HAS-UD Vascular Biology and Myocardial Pathophysiology Research Group, Hungarian Academy of Sciences, University of Debrecen, 4032 Debrecen, Hungary;

[4] Department of Pharmacology and Pharmacotherapy, Faculty of Medicine, University of Debrecen, Nagyerdei krt 98., H-4032 Debrecen, Hungary

[5] Department of Pediatrics, Faculty of Medicine, University of Debrecen, 4032 Debrecen, Hungary

* Correspondence: balla@belklinika.com; Tel.: +36-52-255-500 / 55004

# These authors contributed equally to this work.

A

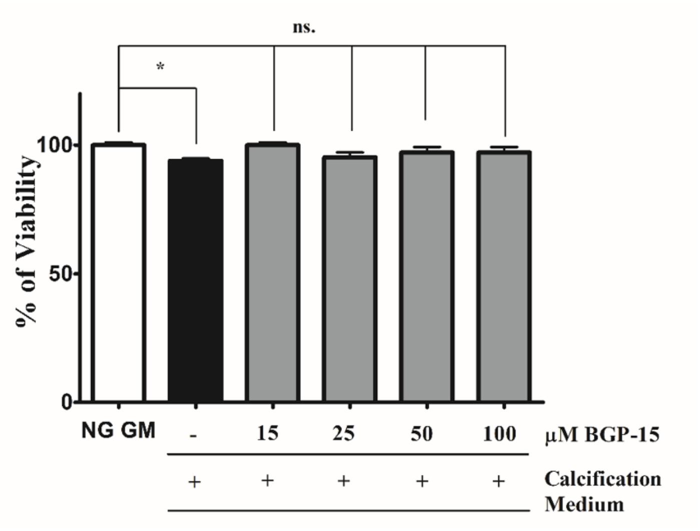

B

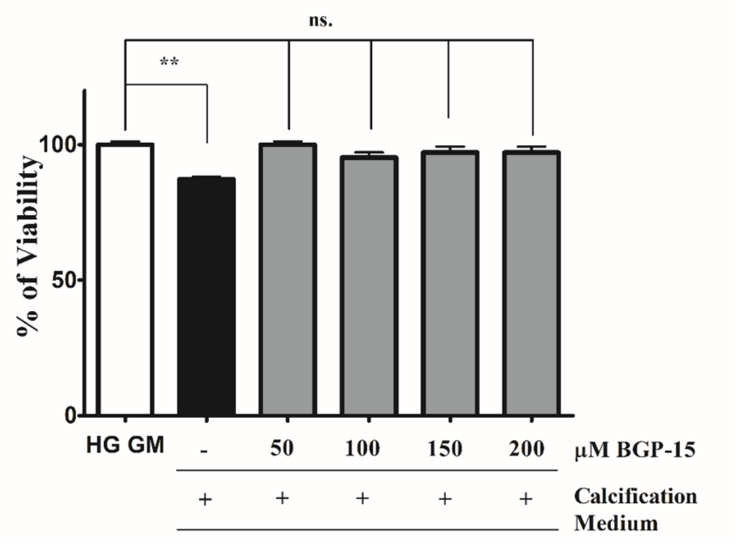

Figure S1. Viability analysis of BGP-15-treated VSMCs. VSMCs were cultured in calcification medium containing 3 mmol/L inorganic Pi under normal glucose (5.5 mM glucose) and high glucose (11 mM) conditions in the presence or absence BGP-15 (15-100 μM BGP-15 in NG and 25-200 μM in HG condition) for 10 days. Cell viability was determined by MTT assay under normal glucose (A) and high glucose (HG) condition. Data are presented as mean ± SEM of three independent experiments. Statistical analysis was performed by one-way ANOVA test followed by Bonferroni correction. A value of $p < 0.05$ was considered significant. *ns*: non-significant; $*p < 0.05$, $**p < 0.01$, $***p < 0.001$.
